# Supplementary material for: Facial Recognition Intensity in Disease Diagnosis Using Automatic Facial Recognition
Source: J Pers Med. 2021 Nov 10;11(11):1172. doi: 10.3390/jpm11111172 (PMC8621146; doi:10.3390/jpm11111172)
Supplement: Supplementary file 1 [file jpm-11-01172-s001.zip › jpm-1435817-supplementary.pdf]

Supplementary Table 1. Search strategy (Medline)

Item	Search terms
1.	exp facial recognition/
2.	(facial adj recognition).mp.
3.	(AI adj fac$).mp.
4.	computer-assisted, face/
5.	(computer adj (assisted or aided) adj fac$).mp.
6.	(automat$ adj fac$).mp.
7.	(machine adj learning adj fac$).mp.
8.	(neural adj network$ adj fac$).mp.
9.	(expert adj system$ adj fac$).mp.
10.	(deep adj learning adj fac$).mp.
11.	(support adj vector adj machine adj fac$).mp.
12.	or/1-11
13	(detect$ or diagnos$ or analy$ or classif$).mp.
14	12 and 13

Supplementary Table 2. Characteristics of included studies

Study	Disease	Data sources	Male (%)	Diagnostic criteria	Control selection	Image resolution	Model/system of machine learning	Sample size of training set	Sample size of testing set	Quality (QUADAS-2)
Basel-Vanagaite 2016a	Cornelia de Lange syndrome	Children's Hospital of Philadelphia, USA	NR	Genes mutation	External, healthy control	NR	FDNA	131	31	Patient selection: unclear; index test: low; reference standard: low; flow and timing: low
Basel-Vanagaite 2016b	Cornelia de Lange syndrome	NR	NR	Genes mutation	External, healthy control	NR	NR	1378	17	Patient selection: unclear; index test: low; reference standard: low; flow and timing: low
Chen 2018	Turner syndrome	PUMCH, Beijing, China	0	Karyotype	Internal, other diseases	640 × 640	SVM	64	64	Patient selection: high; index test: low; reference standard: low; flow and timing: low
E. Miller 2011	Acromegaly	Manchester, UK and Lexington, KY	NR	Previously treatment	External, other diseases	NR	SVM	200	49	Patient selection: high; index test: low; reference standard: unclear; flow and timing: low
Gurovich 2019a	Cornelia de Lange syndrome	Casia-Web-Face	NR	Genes mutation	External, healthy control	100×100	DCNN	1693	32	Patient selection: unclear; index test: low; reference standard: low; flow and timing: unclear

Gurovich 2019b	Angelman syndrome	Casia-Web-Face	NR	Karyotype	External, healthy control	100×100	DCNN	3465	25	Patient selection: unclear; index test: low; reference standard: low; flow and timing: low
Kong 2018	Acromegaly	Several large general hospitals in China and SCUT-FBP	0.518	Growth hormone suppression test	External, other diseases	100×100	LM, KNN, SVM, RT, CNN, and EM	1123	242	Patient selection: high; index test: low; reference standard: low; flow and timing: high
Kong 2020	Acromegaly	CASIAWeb-Face	0.527	NR	External, other diseases	160×160	OpenCV	1911	79	Patient selection: high; index test: low; reference standard: unclear; flow and timing: low
Kosilek 2013	Cushing's syndrome	CASIAWeb-Face	0	Biochemistry	Internal, other diseases	NR	NR	60	60	Patient selection: high; index test: low; reference standard: low; flow and timing: low
Ozdemir 2018a	Fragile X, Hurler, Prader Willi, Down, Wolf Hirschhorn syndromes	Available on the Web pages	NR	NR	Internal, healthy control	155×172	Hierarchical decision tree	130	30	Patient selection: high; index test: low; reference standard: unclear; flow and timing: unclear

Ozdemir 2018b	Fragile X, Hurler, Prader Willi, Down, Wolf Hirschhorn syndromes	Available on the Web pages	NR	NR	Internal, healthy control	155×172	k-NN	130	30	Patient selection: high; index test: low; reference standard: unclear; flow and timing: unclear
Ozdemir 2018c	Fragile X, Hurler, Prader Willi, Down, Wolf Hirschhorn syndromes	Available on the Web pages	NR	NR	Internal, healthy control	155×172	NR	130	30	Patient selection: high; index test: low; reference standard: unclear; flow and timing: unclear
Popp 2019	Cushing's syndrome	Munich and Berlin-Charlottenburg, Germany	0.268	NR	Internal, other diseases	1500 ×1000	FIDA	180	180	Patient selection: high; index test: low; reference standard: low; flow and timing: low
Saraydemir 2012a	Down syndrome	Istanbul, Turkey	0.600	Karyotype	Internal, other diseases	320×240	k-NN	30	30	Patient selection: high; index test: low; reference standard: low; flow and timing: low
Saraydemir 2012b	Down syndrome	Istanbul, Turkey	0.600	Karyotype	Internal, other diseases	320×240	SVM	30	30	Patient selection: high; index test: low; reference standard: low; flow and timing: low

Schneider 2011	Acromegaly	Munich, Germany	0.491	Experts	Internal, other diseases	168×224	NR	116	116	Patient selection: high; index test: low; reference standard: low; flow and timing: low
Valentine 2017	Fetal alcohol spectrum disorders (FASD)	FASER database	NR	Maternal interviews and neuropsychological testing	Internal, other diseases	NR	FDNA	139	139	Patient selection: high; index test: low; reference standard: low; flow and timing: low
Zhao 2013a	Down syndrome	Institutional Review Board (IRB) and Children's National Medical Center.	0.662	NR	Internal, other diseases	512×512	SVM, k-NN,RF,LDA	130	130	Patient selection: high; index test: low; reference standard: low; flow and timing: unclear
Zhao 2013b	14 mixed syndromes	Mixed syndromes dataset	NR	NR	Internal, healthy control	NR	NR	104	104	Patient selection: high; index test: low; reference standard: low; flow and timing: unclear
Pan 2020	Turner syndrome	PUMCH, Beijing, China	0	Karyotype	External, healthy control	224×224	DCNN	1223	37	Patient selection: high; index test: low; reference standard: low; flow and timing: low

NR = not reported.

Supplementary Table 3. Methodological quality assessment of included studies using QUADAS-2

Study	Domain 1a1	Domain 1a2	Domain 1a3	Domain 1b	Domain 2a1	Domain 2a2	Domain 2b	Domain 3a1	Domain 3a2	Domain 3b	Domain 4a1	Domain 4a2	Domain 4a3	Domain 4a4
Basel-Vana-gaite 2016a	unclear	low	low	low	low	low	Low	low	low	low	low	low	low	low
Basel-Vana-gaite 2016b	unclear	low	low	low	low	low	Low	low	low	low	low	low	low	low
Chen 2018	unclear	high	low	low	low	low	Low	low	low	low	low	low	low	low
E. Miller 2011	unclear	high	low	low	low	low	Low	unclear	low	low	low	low	low	low
Gurovich 2019a	unclear	low	low	low	low	low	Low	low	low	low	low	low	low	low
Gurovich 2019b	unclear	low	low	low	low	low	Low	low	low	low	low	low	low	low
Kong 2018	unclear	high	low	low	low	low	Low	low	low	low	low	low	high	low
Kong 2020	unclear	low	low	low	low	low	Low	low	low	unclear	low	low	low	low
Kosilek 2013	unclear	high	high	low	low	low	low	low	low	low	low	low	low	low
Ozdemir 2018a	unclear	low	low	high	low	low	low	unclear	low	low	low	unclear	low	low
Ozdemir 2018b	unclear	low	low	high	low	low	low	unclear	low	low	low	unclear	low	low
Ozdemir 2018c	unclear	low	low	high	low	low	low	unclear	low	low	low	unclear	low	low
Popp 2019	unclear	high	low	low	low	low	low	low	low	low	low	low	low	low
Saraydemir 2012a	unclear	high	low	low	low	low	low	low	low	low	low	low	low	low
Saraydemir 2012b	unclear	high	low	low	low	low	low	low	low	low	low	low	low	low
Schneider 2011	unclear	high	low	low	low	low	low	low	low	low	low	low	low	low

Valentine 2017	unclear	high	low	low	low	low	low	low	low	low	low	low	low	low
Zhao 2013a	unclear	high	low	low	low	low	low	low	low	low	low	low	low	low
Zhao 2013b	unclear	high	low	low	low	low	low	low	low	low	low	low	low	low
Pan 2020	unclear	high	low	low	low	low	low	low	low	low	low	low	low	low
